# Meteorological Report for November

**Published:** 1878-12

**Authors:** George H. Rohe

**Affiliations:** U. S. Signal Service


### METEOROLOGICAL REPORT FOR NOVEMBER.

(Furnished by Dr. George H. Rohe, U. S. Signal Service.)

| DATE. 1878. | Mean daily Barometer. | Mean daily Thermometer | Mean daily Humidity. | Maximum Thermometer | Minimum Thermometer | Prevailing direction. | Daily Rainfall. |
|---|---|---|---|---|---|---|---|
| 1 | 30.304 | 45.7 | 43.0 | 54 | 30 | N. W. | |
| 2 | 30.247 | 53.0 | 42.0 | 62 | 40 | W. | |
| 3 | 30.229 | 55.0 | 46.3 | 64 | 46 | N. W. | |
| 4 | 30.214 | 54.5 | 49.3 | 65 | 42 | N. W. | |
| 5 | 30.199 | 56.7 | 57.0 | 64 | 44 | E. | |
| 6 | 30.083 | 60.7 | 64.7 | 69 | 46 | S. W. | |
| 7 | 29.994 | 63.7 | 67.3 | 71 | 59 | W. | .01 |
| 8 | 30,043 | 54.2 | 53.3 | 63 | 49 | N. W. | |
| 9 | 30.111 | 52.7 | 53.0 | 61 | 43 | N. W. | |
| 10 | 30.044 | 54.7 | 56.3 | 65 | 42 | S. E. | |
| 11 | 29.783 | 61.0 | 68.3 | 68 | 51 | S. W. | .04 |
| 12 | 29.936 | 51.2 | 69.0 | 58 | 46 | N. W. | |
| 13 | 30.043 | 53.7 | 49.7 | 63 | 41 | N. W. | |
| 14 | 30.206 | 54.0 | 45.3 | 62.5 | 45 | E. | |
| 15 | 30.183 | 46.5 | 64.3 | 51 | 44 | E. | .13 |
| 16 | 30.033 | 51.0 | 93.0 | 54 | 46 | E. | .46 |
| 17 | 30.076 | 56.0 | 85.3 | 64 | 50 | N. W. | |
| 18 | 29.979 | 58.5 | 71.3 | 66 | 51 | N. W. | |
| 19 | 29.814 | 52.5 | 88.3 | 55 | 50 | N. E. | .09 |
| 20 | 29.825 | 53.5 | 75.3 | 61 | 46 | N. W. | |
| 21 | 29.731 | 47.0 | 74.7 | 54 | 46 | N. W. | .48 |
| 22 | 29.756 | 45.0 | 54 3 | 52 | 38 | N. W. | |
| 23 | 29.867 | 55.0 | 47.0 | 62 | 41 | N. W. | |
| 24 | 30.007 | 59.7 | 74.0 | 67 | 47 | S. W. | |
| 25 | 29.978 | 63.0 | 85.7 | 70 | 59 | S. | .12 |
| 26 | 29.802 | 62.5 | 94.0 | 65 | 59 | S. E. | 1.22 |
| 27 | 29.792 | 45.7 | 76.3 | 63 | 40 | S. W. | 1.99 |
| 28 | 30.133 | 42.7 | 67.3 | 50 | 35 | N. W. | |
| 29 | 30.232 | 49.0 | 63.3 | 58 | 34 | N. W. | |
| 30 | 30 228 | 50.7 | 59.0 | 57 | 42 | E. | |
| 31 | ......... | ...... | ..,... | ... | ... | ......... | ...... |
| Sums..... | ........... | ........... | ........... | ......... | ......... | ............... | 4.54 |
| Monthly Means. | 30.029 | 53.6 | 64.6 | 61.3 | 45.1 | N. W. | |

Highest Barometer, 30.394 on the 1st.
Lowest Barometer, 29.638, on the 21st.
Monthly range of Barometer, 756.
Highest Temperature, 71°, on 7th. Lowest Temperature, 30° on 1st.
Monthly range of Temperature, 41°.
Greatest daily range of Temperature, 24° on the 1st and 29th.
Least daily range of Temperature, 5°, on the 19th.
Total rainfall, 4.54 inches.
Depth of unmelted snow lying on ground at end of month, — inches.
Prevailing wind, Northwest. Total movement of wind, 79.31 miles.
Maximum velocity of wind and direction, 31 miles, Southwest, on 27th.
*Number of Foggy days, none. Number of Clear days, 13.
Mumber of fair days, 8. Number of cloudy days on which rain fell, 6.
Number of cloudy days on which no rain fell, 3.
Total number of days on which rain fell, 9.

